# Spatial and temporal connectivity of brain resting-state fMRI during music-induced analgesia in fibromyalgia patients

**DOI:** 10.3389/fnhum.2026.1622082

**Published:** 2026-05-05

**Authors:** Jiancheng Hou, Ying Liu, Thomas Hosseini, Maoping Zheng, Xiaolin Liu, Changan Sun

**Affiliations:** 1 Research Center for Cross-Straits Cultural Development, Fujian Normal University, Fuzhou, Fujian, China; 2 Department of Radiology, School of Medicine and Public Health, University of Wisconsin-Madison, Madison, WI, United States; 3 Academy of Music, Southwest University, Chongqing, China; 4 School of Education, Suzhou University of Science and Technology, Suzhou, Jiangsu, China

**Keywords:** fibromyalgia, music-induced analgesia, fractional amplitude of low frequency fluctuations, regional homogeneity, degree centrality

## Abstract

**Introduction:**

Music-induced analgesia (MIA) has significant clinical value for patients with fibromyalgia (FM) and serves as a key model for understanding the complex neural mechanisms underlying the effects of music on physical and mental states. However, previous research offers limited interpretation of the broader neural network characteristics underlying pain regulation through music, particularly regarding spatial synchronization and temporal connectivity.

**Methods:**

The present study examined the neural correlates of MIA in FM patients using resting-state functional magnetic resonance imaging (RS-fMRI) with fractional amplitude of low frequency fluctuations (fALFF), regional homogeneity (ReHo) and degree centrality (DC) measures. Twenty female FM patients underwent RS-fMRI scans before and after listening to self-selected, familiar, highly pleasant, and slow-tempo music. Behavioral assessments of pain intensity (PI) and pain unpleasantness (PU) were collected immediately before and after music listening. Paired t-tests and correlation analyses were performed.

**Results:**

Following music listening, FM patients exhibited a significant reduction in PI and a marginal reduction in PU. Significant RS-fMRI changes were observed: increased fALFF in the frontal, occipital, and cerebellar regions; increased ReHo in the precentral gyrus, cerebellum_crus2, and postcentral gyrus; decreased ReHo in the temporal, occipital, frontal, limbic, and cingulate regions; and increased DC in frontal areas and the supplementary motor area. Additionally, significant correlations were found between clinical measures and fALFF or ReHo measures, including positive correlations between PI and fALFF in the left middle occipital gyrus, and negative correlations between PU and fALFF/ReHo in the right cerebellum_crus2 and left middle occipital gyrus.

**Conclusion:**

These findings suggested the effectiveness of music’s pain regulation function in FM patients, potentially through both cognitive and emotional pathways. The spatial and temporal fMRI evidence highlights key roles of the frontal, occipital, sensorimotor, limbic, and cerebellar regions in MIA, providing a more comprehensive framework for understanding its underlying neural mechanisms.

## Introduction

Music has evolved beyond its traditional artistic role to encompass diverse physical and mental health benefits, including psychological adjustment, social value guidance, medical applications, and artificial intelligence integration (Becker et al., 2024; Finn and Fancourt, 2018). In clinical settings, music not only serves as an effective analgesic during procedures, but also plays a key role in postoperative physical and mental rehabilitation. Music-induced analgesia (MIA) has been widely applied in the treatment of various conditions, demonstrating efficacy in both experimental pain models in healthy individuals (Choi et al., 2018) and in patients experiencing acute (Cakmak et al., 2017; Simavli et al., 2014), postoperative (Hole et al., 2015), and chronic pain (Garza-Villarreal et al., 2017).

Research suggests that MIA primarily operates through cognitive and emotional regulatory mechanisms (Tracey and Mantyh, 2007). Cognitive factors such as distraction and attention contribute to music’s analgesic effects (Basinski et al., 2021; Chang et al., 2015). While no studies have directly mapped cognitive function-specific brain regions in MIA, research on music therapy highlights the role of the fronto-parietal, dorsal attention, salience, and default mode networks in cognitive modulation (Powers et al., 2022). Emotion regulation also plays a key role in MIA, particularly through the pain-relieving effects of pleasurable or participant-familiar music (Maidhof et al., 2025; Roy et al., 2008; Valevicius et al., 2023), which involves reward-related brain regions such as the limbic system, frontal cortex, and auditory regions (Dobek et al., 2014; Porreca and Navratilova, 2017; Powers et al., 2022).

Given the increasing recognition of music’s physical and psychological effects, understanding the neural mechanisms underlying MIA is crucial: neural evidence can help elucidate how music modulates pain through both cognitive and emotional pathways. One study found that preferred music significantly lowered pain ratings in response to acute painful stimuli compared to disliked or no music (Lu et al., 2023). These reductions were linked to activation in sensory pain-processing regions such as the right precentral and postcentral gyri (PreCG/PoCG), which are related to affective components of pain; the anterior cingulate cortex and bilateral putamen, which are associated with motor control and avoidance reactions to pain; and the left cerebellum, which is involved in motor balance and balance control (Lu et al., 2023). The study also showed that pain relief under different music conditions was mediated by activation in the right PreCG/PoCG and left cerebellum (Lu et al., 2023). This evidence suggests that MIA involves the integration of multiple brain regions in spatial synchronization (Werner et al., 2023). Additionally, temporal variations in blood oxygen level-dependent responses play an important role in explaining the pain modulation effects of music. Powers et al. (2022) used Bayesian regression to analyze individual differences in pain perception and found that music enhanced predictive and reactive responses to noxious stimuli in the insula and thalamus, suggesting a predominantly sensory/discriminative signaling effect in MIA.

As a non-invasive pain intervention, MIA has been applied to the treatment of fibromyalgia (FM), a disease that accompanies typical chronic pain (Finn and Fancourt, 2018). FM is presented as much as 2 to 8% in population, is characterized by widespread pain, and is often accompanied by fatigue, memory problems and sleep disturbances (Clauw, 2014). When investigating the neural correlates of MIA in FM patients, Pando-Naude et al. (2019) found that FM patients reported MIA that was significantly correlated with resting-state functional connectivity (RSFC) decreases between the angular gyrus, posterior cingulate cortex and precuneus, and RSFC increase between the amygdala and middle frontal gyrus. These areas are related to autobiographical and limbic processes, and auditory attention, suggesting MIA may arise as a consequence of top-down modulation, probably originated by distraction, relaxation, positive emotion, or a combination of these mechanisms.

While previous research has provided insight into the functional neural activity of brain regions involved in MIA, it offers limited interpretation of the broader neural network characteristics underlying pain regulation through music. One reason is that many studies have conducted the seed-based correlation analyses, which is a way of examining known brain regions and limits the judgment of neural involvement in whole brain. On the other hand, existing studies lack the analysis of temporal and spatial characteristics of data, thus constraining functional interpretations of brain activity. A more comprehensive understanding of MIA requires examination of both spatial synchronization and temporal connectivity to better capture its neural mechanisms.

RSFC has traditionally been used to study cerebral activations, but suffers from certain limitations. RSFC relies on predefined region of interests (ROIs) to analyze linear correlations or mean time-series of each voxel (Tian et al., 2012). This approach does not provide adequate information regarding localized brain activity and spontaneous neural fluctuations within individual regions (Liu et al., 2010), making the results highly dependent on ROI selection (Smith et al., 2011; Tian et al., 2012). To address these limitations, several alternative methods focus on local measures to complement the insufficiency of the RSFC approach. One such local factors is the amplitude of low frequency fluctuations (ALFF), which evaluates the amplitude of spontaneous fluctuations in certain voxels (Zang et al., 2007; Zou et al., 2008). The fractional ALFF (fALFF) improves accuracy by normalizing these fluctuations within a specific frequency band by measuring the ratio of the power spectrum of the low-frequency range to that of the whole frequency range (Zou et al., 2008), thus reducing biases from physiological noise (Lai and Wu, 2012; Zou et al., 2008). Regional homogeneity (ReHo) examines the synchronization or similarity of time series around neighboring voxels, providing insight into localized temporal coordination (Liu et al., 2010; Zang et al., 2004). Additionally, degree centrality (DC), a graph theory-based measure, quantifies the number and strength of network connections for each voxel, identifying key hubs in whole-brain networks with high sensitivity (Li et al., 2016; Zhang et al., 2019; Zuo et al., 2012). Unlike RSFC, these four measures do not require predefined ROIs and can provide information about the local activity of separate brain regions (Tian et al., 2012).

Given their advantages in capturing spatial synchronization and temporal variations, the current study employs resting-state functional MRI (RS-fMRI) with ALFF, fALFF, ReHo and DC to investigate functional differences in MIA in FM patients. This approach enhances the interpretation of MIA’s neural characteristics and provides a more comprehensive framework for understanding its underlying mechanisms.

## Methods

### Data source

Participant data, including resting-state functional MRI (RS-fMRI) and anatomical T1-weighted images, were obtained from the publicly available OpenNeuro dataset (accession number: ds001928).^1^ The study included 20 female fibromyalgia (FM) patients (mean age: 46.4 ± 12.4 years, range: 22–70 years). Clinical information could be assessed in Table 2 of Pando-Naude et al. (2019) study. Ethical approval was granted by the Bioethics Committee of the Instituto de Neurobiología, UNAM. Inclusion and exclusion criteria followed those outlined by Pando-Naude et al. (2019).

### Stimulus materials

Prior to the study, participants provided a list of songs or artists that they would like to listen during the experiment. The Songs were familiar, very pleasant, and slow. The slow pace was defined as a tempo of <120 beats per minute (bpm), determined by the experimenters using a metronome. Participants reported how pleasant the song was on a 11-point verbal scale (0 = unpleasant, 10 = very pleasant), and to be selected, the song had to be rated at least 9/10. When provided with only the artist’s name, the experimenter selected the songs based on two fixed acoustic criteria: harmony (pleasurable), participants’ oral reporting, and slow tempo. Based on a previous study, music-induced analgesia for chronic pain was higher when participants chose familiar and happy music, making self-selection and familiarity an important mechanism for this effect (Pando-Naude et al., 2019).

### Design and paradigm

Before the experiment, all participants were briefed about the study to make sure they understood the procedure and implications, including the MRI scan. Participants then answered the behavioral questionnaires described below. For the auditory stimuli, the NordicNeuroLab AS (Bergen, Norway) MRI safety headphones were used, and their presenting orders were balanced among the participants in order to avoid sequential effects. Each participant underwent two RS-fMRI acquisitions that were performed before (pre-) and after (post-) music listening, each lasting 5 min. Inside the MRI scanner, participants listened to a 5-min segment of familiar, slow paced, and highly pleasant music (presented before and after the brain scan). No imaging was recorded during the auditory stimulation period. Before the RS-fMRI acquisition, the anatomical T1-weighted data were acquired (Pando-Naude et al., 2019). Meantime, two behavioral tasks, the pain intensity (PI) and the pain unpleasantness (PU), a type of Likert scale commonly employed in pain research, were tested while the FM patients were in the MRI scanner and using an 11-point verbal rating scale (0 = no pain, 10 = worst pain possible). PI measures the sensory dimension of pain, and the PU measures the evaluative and emotional dimension of pain (Pando-Naude et al., 2019). The two tasks were performed immediately before and after the music stimuli.

It should be noted that the original study employed a block-based resting-state design rather than a continuous task-based paradigm. The BOLD signals analyzed were derived from the resting-state fMRI scans conducted before and after music listening, not during the music stimulation itself. Consequently, the analysis focused on changes in resting-state functional connectivity between the pre- and post-music blocks, rather than on event-related responses during music exposure. In the present study, utilizing the same dataset, we similarly analyzed the pre- and post-music resting-state blocks. However, we employed local measures—ALFF, fALFF, ReHo, and DC—which are computed from the continuous resting-state BOLD time series within each block. This approach allows for the examination of spatially localized and temporally coordinated neural activity changes associated with MIA.

### MRI data acquisition

The original neuroimaging dataset was acquired on a 3.0 Tesla GE Discovery MR750 scanner (HD, General Electric Healthcare, Waukesha, WI, USA) and a commercial 32-channel head coil array. The parameters for RS-fMRI NIFTI data were: TR = 3,000 ms, TE = 40 ms, flip angle = 90°, field of view = 256 × 256 mm^2^, voxel size = 2 × 2 mm, number of slices = 43, matrix = 128 × 128, slice thickness = 3 mm, gap = 0 mm. The parameters for anatomical T1-weighted NIFTI data were: TR = 7.7 ms, TE = 3.2 ms, flip angle = 12°, field of view = 256 × 256 mm^2^, matrix = 256 × 256, slice thickness = 1 mm, number of slices = 168, gap = 0 mm.

### Data preprocessing

Preprocessing of the RS-fMRI data was performed using the Data Processing Assistant for Resting-state fMRI Advanced Edition toolbox (DPARSF V5.3), which is part of the Data Processing and Analysis of Brain Imaging (DPABI) toolbox version 6.0^2^ (Chang and Glover, 2010; Chao-Gan and Yu-Feng, 2010; Yan et al., 2016). DPARSF is a convenient plug-in software based on Statistical Parametric Mapping (SPM, version 12)^3^ and Resting-State fMRI Data Analysis Toolkit (RESTplus V1.24)^4^ is integrated in Matlab (Chao-Gan and Yu-Feng, 2010). The Digital Imaging and Communications in Medicine (DICOM) files were first arranged and the parameters, such as repeating time, slice number, voxel size, were then set. DPARSF then produced the preprocessed data, including slice timing, realignment, and normalization. In detail, the first five volumes were discarded to allow the magnetization to approach a dynamic equilibrium, and for the participants to get used to the scanner noise; the following steps were slice timing (to eliminate the errors caused by technical or physiological factors in the process of image acquisition, and ensure the accuracy and comparability of data), realignment (to reduce motion interference and ensure the authenticity of time series signals‌), scrubbing within Friston 24-parameter model regression (to remove the nuisance signals and also to regress out head motion effects from the realigned data) (Friston et al., 1996); spatial normalization to the Montreal Neurological Institute (MNI) template (resampling voxel size of 3 × 3 × 3 mm) (to ensure that the data distribution is as consistent as possible, so that the intensity value of the image falls within a specific interval); band-pass filtering (with frequency of 0.01–0.1 Hz) (to reduce the effects of low-frequency drift and high-frequency physiological noise) (Chao-Gan and Yu-Feng, 2010; Kuhn et al., 2012; Leonardi and Van De Ville, 2015; Wang et al., 2018; Zhou et al., 2019). Band-pass temporal filtering (0.01–0.1 Hz) was applied for ReHo and DC analyses, but was omitted for ALFF and fALFF. The ALFF, fALFF, ReHo and DC measures were then calculated using Fisher *z*-transformation, followed by spatial smoothing with a 4-mm full-width at half maximum (FWHM) Gaussian filter.

### Statistical analysis

The statistical analysis was also performed in DPABI. The paired *t*-test (after vs. before music listening) was conducted to compare ALFF, fALFF, ReHo, and DC differences between the post- vs. pre-music listening in FM patients. Multiple comparison correction was completed using Threshold-Free Cluster Enhancement (TFCE) with 5,000 permutations (Smith and Nichols, 2009) for all four measures. A significance threshold of TFCE *p* < 0.05 and cluster size >100 was applied. Finally, DPABIViewer was used to generate visual representations of the results.

In order to perform the correlation analysis, we firstly extracted the brain signals both in post- and pre-music listening, respectively, in the ALFF (or the fALFF, ReHo, DC) from the significant clusters (through the coordinates from the peak *t* vales in Table 1) through the ROI Signal Extractor utility in DPABI; then the subsequent correlation analysis between the behavioral PI task (or the PU task; the difference value between post- minus pre-music listening) and the ALFF (or the fALFF, ReHo, DC, respectively; the difference value between post- minus pre-music listening) was computed by Pearson’s correlation with a threshold of *p* < 0.05 with SPSS version 23.

**Table 1:** RS-fMRI contrasts between post- vs. pre-music listening.

Measures	Regions (BA)	Peak coordinates (MNI)			Peak *t* value	Cluster size
		*x*	*y*	*Z*		
fALFF	R middle frontal gyrus (48)	36	33	21	5.307	3,712
	R calcarine (18)	18	−96	0	4.131	258
	R cerebellum_crus2	45	−72	−48	−2.945	137
	L middle occipital gyrus (19)	−36	−84	−3	3.628	217
	L lingual gyrus (18)	−9	−63	0	4.200	761
	L superior frontal gyrus (9)	−24	36	60	3.367	302
ReHo	R precentral gyrus (3)	39	−18	39	3.290	213
	R cerebellum_crus2	9	−81	−33	5.226	165
	R superior temporal gyrus (21)	60	−57	21	−4.210	305
	R pallidum	24	−3	3	−5.313	936
	R middle occipital gyrus (19)	42	−90	12	−4.089	159
	R middle cingulate cortex (32)	9	9	42	−5.021	5,465
	R middle frontal gyrus (46)	51	42	16	−3.496	365
	R middle temporal gyrus (21)	60	−6	−18	−3.659	360
	L postcentral gyrus (3)	−27	−30	48	3.543	214
	L parahippocampus (28)	−12	3	−27	−3.929	218
	L middle occipital gyrus (19)	−33	−78	21	−3.122	187
DC	R middle frontal gyrus (45)	45	45	18	3.905	245
	R middle temporal gyrus (21)	57	−3	−24	−4.003	269
	L superior medial frontal gyrus (9)	−9	39	48	3.199	136
	L superior frontal gyrus (6)	−18	17	67	4.159	100
	L supplementary motor area (6)	0	−6	75	3.637	145

The multiple comparison correction was used with non-parametric permutations (*n* = 5,000) and threshold-free cluster enhancement (TFCE) correction *p* < 0.05, cluster size >100. BA: Brodmann area; MNI: Montreal Neurological Institute template; L: left; R: right. Positive *t* value: post- > pre-music listening; negative *t* value: post- < pre-music listening.

## Results

### Behavioral tests

The paired *t*-test showed that after music listening, the FM patients’ PI was significantly decreased, and the PU was marginally decreased (see Table 2), compared to before music listening.

**Table 2:** Behavioral tasks between post- vs. pre-music listening.

Tasks	Post-music listening	Pre-music listening	*t*_(19)_	*p*
PI	3.350 (3.249)	4.300 (3.011)	−2.967	0.008
PU	3.700 (3.481)	4.500 (3.137)	−1.775	0.092

PI: pain intensity; PU: pain unpleasantness. The values in parentheses represent the standard deviation.

### RS-fMRI contrasts between post- vs. pre-music listening

**Figure 1:**
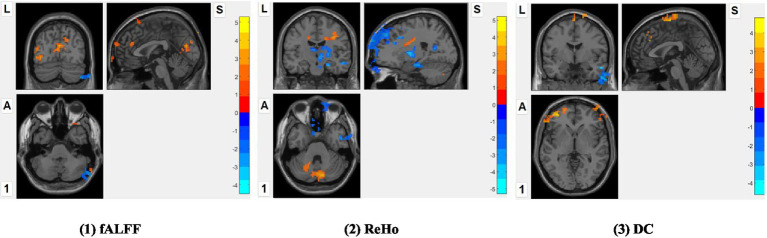
RS-fMRI contrasts between post- vs. pre-music listening. The multiple comparison correction was used with non-parametric permutations (*n* = 5,000) and threshold-free cluster enhancement (TFCE) correction *p* < 0.05, cluster size >100. L: Left; A: anterior; S: superior. Red color: post- > pre-music listening; blue color: post- < pre-music listening.

Compared to pre-music listening, post-music listening induced: (1) increased fALFF in the right middle frontal, calcarine gyri, left middle occipital, lingual, superior frontal gyri, but decreased fALFF in the right cerebellum_crus2; (2) increased ReHo in the right precentral gyrus, cerebellum_crus2, left postcentral gyrus, but decreased ReHo in the right superior temporal, middle occipital, middle frontal, middle temporal gyri, pallidum, middle cingulate cortex, left parahippocampus and middle occipital gyrus; (3) increased DCin the right middle frontal gyrus, left superior medial frontal, superior frontal gyri, supplementary motor area, but decreased degree centrality in the right middle temporal gyrus (see Table 1 and Figure 1). Nevertheless, no significant ALFF difference between post- vs. pre-music listening.

**Table 3:** Correlations between behavioral tasks and RS-fMRI measures.

Measures	Regions	Tasks	*r*_(20)_	*p*
fALFF	Right cerebellum_crus2	PU	−0.454	0.044
	Left middle occipital gyrus	PI	0.481	0.032
ReHo	Left middle occipital gyrus	PU	−0.474	0.042

PI: pain intensity; PU: pain unpleasantness.

### Correlative analysis

In fALFF, there were significant correlations between the PU had significantly negative correlation to the right cerebellum_crus2, and PI had significantly positive correlation to the left middle occipital gyrus. In ReHo, the PU had significantly negative correlation to the left middle occipital gyrus (see Table 3).

## Discussion

The current study investigated the neural correlates of MIA in FM patients using RS-fMRI with fALFF, ReHo and DC measures based on the data-driven analysis of RS-fMRI. Following music listening, FM patients exhibited significant reduction in evaluation of pain intensity, and also exhibited significant RS-fMRI changes in fALFF, ReHo, and DC, which mainly occurred in the frontal, occipital, central areas, limbic system and cerebellum. Additionally, correlations were found between behavioral task and fALFF or ReHo measure. These findings suggest the effectiveness of music’s pain regulation function and provide spatial and temporal evidence for understanding the neural mechanism underlying MIA.

Behavioral results demonstrated the effective regulation of music on pain perception in FM patients. Specifically, the pain intensity level in FM patients changed significantly after the music listening, and the pain unpleasure also decreased but did not reach significant level. The results indicate that music may independently regulate pain perception and pain emotion; that is, the generation of MIA may both involve cognitive processing and emotional regulation, which can be explained by a general framework of three factors affecting pain perception as elaborated by Tracey and Mantyh (2007), who also primarily proposed three aspects of MIA: cognition, emotion, and neurobiology (Lunde et al., 2019). The cognitive-factor is often discussed in regards to MIA is distraction and attention, which can situate the analgesic capacity of music on a cognitive level (Basinski et al., 2021; Chang et al., 2015). The emotion-factor underscores that music-induced emotion is a key mechanism, which functions in pleasure enhancement and anxiety reduction (Roy et al., 2008; Valevicius et al., 2023). When participants listen to their favorite music compared with no music during painful stimulation, fMRI studies have demonstrated different activation in several areas of the limbic system and areas known to be involved in the descending pain-modulatory system (Dobek et al., 2014) and brain functional connectivity across these neural networks between regions such as the insula, thalamus, hypothalamus, amygdala and hippocampus (Powers et al., 2022). Meanwhile, when exploring the cognitive mechanisms in music listening or music interventions for pain, the *Cognitive Vitality Model* illustrates that MIA happens with five themes: automated attention, cognitive agency, meaning-making and enjoyment, musical integration, and cognitive vitality (Howlin and Rooney, 2020). From these two theoretical models it can be speculated that the difference between PI and PU was formed from the gradual emergence of cognitive processing at different levels and different themes.

The potential independent processing between PI and PU can also be reflected in the correlations of behavioral tasks and RS-fMRI measures. In current study, a positive correlation was found between PI and fALFF in the left middle occipital gyrus, but negative correlations were found between PU and fALFF in the right cerebellum_crus2, PU and ReHo in the left middle occipital gyrus. During music processing, the left middle occipital gyrus has been found tightly connected with visual attention, harmonic processing, and viusal imagination (Cai et al., 2018; Sato et al., 2015; Tanaka and Kirino, 2019). In further detail, fALFF evaluates the fractional amplitude of spontaneous fluctuations in certain voxels but ReHo examines the synchronization or similarity of time series around neighboring voxels. The opposite correlation trend between the left middle occipital gyrus and PI or PU indicates that the left middle occipital gyrus may increase the fractional amplitude of spontaneous fluctuations and decrease synchronization of time series around neighboring voxels to generate positive pain management.

When compared the RS-fMRI between post- and pre-music listening, significant results were found by measuring fALFF, ReHo and DC. From the distribution characteristics of brain regions in fALFF, the amplitude of spontaneous fluctuations mainly increased in the middle and superior frontal gyri, middle occipital gyrus and decreased in the right cerebellum_crus2. Dobek et al. (2014) found that frontal regions are the important brain regions related to pleasant music listening. In current study, patients were guided to listen to their familiar, very pleasant and slow songs. Combined with the visual processing function of the middle occipital gyrus (Cai et al., 2018; Sato et al., 2015; Tanaka and Kirino, 2019), the increased frontal and occipital regions here indicate that MIA may dependent on evoking patients’ positive emotion and visual processing to realize the effect of pain relief.

As for the decreased fALFF in the right cerebellum_crus2, it is different from previous results that the enhanced activity was found in the right cerebellum (Li et al., 2019; Pando-Naude et al., 2019). The cerebellum has been found to be an important brain region involved in the processing of music-related autobiographical memory (Falcon et al., 2022; Lesiuk et al., 2024) and attentional modulation (Wang et al., 2025). The cerebellum_crus2 was found specialized for social mentalizing and emotional self-experiences (Moe et al., 2025). In current study, whether the reduction of spontaneous fluctuations in the cerebellum is related to the inhibitory patients’ previous experience of pain memory or pain emotion deserves further study. What’s more, it is worth noting that the enhanced activation found in previous studies was mainly in the anterior part of cerecbellum. In current study, the decreased fALFF in the cerebellum was found more in its posterior part. Whether there are regional differences for pain management in the cerebellum, such as the anterior part for managing emotions and the posterior part for managing cognition (e.g., attention and memory), is also worthy of further study.

For the result of ReHo, significant increased ReHo was found in the right precentral gyrus, cerebellum_crus2, left postcentral gyrus, but decreased ReHo in the right superior temporal, middle occipital, middle frontal, middle temporal gyri, pallidum, middle cingulate cortex, left parahippocampus and middle occipital gyri. The precentral gyrus is the center of body movement in humans and an important structure for executing voluntary movements (Rivara et al., 2003). The postcentral gyrus is the somatosensory and motor center and is mainly responsible for the regulation of movement and the maintenance of posture (Borich et al., 2015). The increased ReHo in the right precentral gyrus suggests that the functional activity of the somatosensory cortex is increased after music listening (Yin et al., 2024). In current study, the increased ReHo of the right precentral gyrus, cerebellum_crus2, and left postcentral gyrus indicates that music listening may enhance the temporal coordination between the somatosensory system and cerebellum, with enhanced somatosensory/sensory experience and emotional self-experiences, to relieve listeners’ pain experience.

Meanwhile, decreased ReHo in the right superior temporal, middle occipital, middle frontal, middle temporal gyri, pallidum, middle cingulate cortex, left parahippocampus, and middle occipital gyrus were also found in current study, which are mainly involved in the limbic system, occipital and auditory regions. In previous studies, the cingulate cortex, parahippocampus, and superior/middle temporal gyri were found to be tightly connected with music-evoked emotion (Khalfa et al., 2008; Koelsch, 2010; Koelsch et al., 2006; Koelsch and Siebel, 2005) and emotion regulation (Moore, 2013). When music modulated the pain perception, the cingulate cortex, parahippocampus and superior/middle temporal gyri were found more activated compared with pain-only condition (Dobek et al., 2014), which supposed that the brain regions associated with Reho in this study may have been involved in the emotional regulation process of MIA. The emergence of MIA also relies on the basic temporal law of music processing and the decreased Reho in above regions is actually a necessary process for patients to achieve MIA after music listening. By incorporating with the time course of brain activity in the limbic system, occipital regions, and auditory regions, Koelsch and Siebel (2005) laid out a model of music perception temporally involving early processing stages, intelligence participating stage, and body reacting stage, which indicates that the temporal dynamics of music perception would reduce the temporal synchrony among different brain regions. In the Cognitive Vitality Model, Howlin and Rooney (2020) also explained the listening schedules of MIA with five themes evolving from low-level attention to high-level cognitive capacity of self-enhanced motivation. These theories inspire us that the decreased Reho in current study may reflect that music listening may achieve patients’ processing of music (which inherently has typical temporal dynamic stimuli) by reducing the temporal synchrony of the right superior temporal, middle occipital, middle frontal, middle temporal gyri, pallidum, middle cingulate cortex, left parahippocampus and middle occipital gyri to participate in MIA.

For the DC results, significant enhancement was found in the frontal area (left superior frontal, superior medial frontal, and right middle frontal gyri) and supplementary motor area. As DC reflects key spatial synchronization connections across the whole brain, the significantly increased DC here suggests that after music listening, FM patients have more efficient neural connectivity compared to before music listening.

### Limitations and future research

By analyzing a publicly available imaging dataset of FM participants before and after music listening, the current study yielded some potentially interesting RS-fMRI results. However, some limitations need to be addressed. First, the study did not design steps for independently detecting PI and PU, which limited the verification of the possible independence of these two mental processing methods.

Second, some typical left–right brain lateralization differences appeared in current study, but they were not enough to be reflected in the lateralization analysis. The lateralization of brain function during music processing has long been debated in neuroscience (Zatorre, 2022), raising questions about whether music processing lateralization truly exists, how it compares to language lateralization, and whether it depends on specific musical elements such as semantics, pitch or intervals. Future research should further differentiate the lateralized effects of MIA in order to better exert the psychosomatic therapeutic value of music.

Third, the study did not incorporate a formal MRI habituation or acclimatization protocol (e.g., mock scanner session) prior to scanning. FM patients are known to exhibit heightened sensitivity to sensory inputs, and the MRI environment itself may induce anxiety, discomfort, or stress, which could potentially confound baseline (pre-music listening) pain ratings and resting-state neural measures. Although participants received a pre-scan briefing and listened to self-selected pleasant music—which may have promoted relaxation and positive affect—the absence of systematic habitation remains a methodological limitation. Future neuroimaging studies involving chronic pain populations should consider implementing standardized acclimation procedures to better isolate disease-specific neural signatures from state-dependent responses to the scanning context.

Fourth, although the current study did not explore the neurochemical mechanisms of MIA, we believe that music can induce analgesia by affecting neurotransmitters such as dopamine, serotonin and endorphins, which are involved in pleasure, pain regulation and mood regulation. For example, music (especially the type of music that evokes a strong emotional response) can trigger the releases of dopamine, a neurotransmitter associated with pleasure and reward that promotes the experiences of pleasure and well-being and reduces the perception of pain (Mallik et al., 2017). Music can also reduce pain and anxiety by affecting the levels of serotonin, which plays a role in mood regulation, sleep, and pain perception (Garza-Villarreal et al., 2017). Moreover, music can release the endorphins, which can increase pain tolerance and happiness (Garza-Villarreal et al., 2017). Based on the current study, we would further perform an original study that integrate more comprehensive participant information, music information, and brain activity evidence to further explore the neurochemical mechanisms of MIA on chronic pain and provide more effective evidences to clinical practice.

Fifth, the clinical heterogeneity of the FM sample—including variations in medication use, sleep quality, and daily fluctuations in anxiety and depression—was not systematically captured in the available dataset. While the original study included the measures of depression (the Center for Epidemiologic Studies Depression Scale) and anxiety (the State–Trait Anxiety Inventory), day-specific state measures, detailed medication logs, and objective sleep assessments were not recorded. These factors may influence both baseline pain perception and responsiveness to music interventions, and their absence limits our ability to control for potential confounds or examine subgroup responses. Future neuroimaging studies in chronic pain populations should incorporate more granular clinical phenotyping—such as sleep diaries, medication registers, and ecological momentary assessment of mood—to better characterize individual differences in treatment response and to isolate music-specific neural effects from state-dependent variability.

Sixth, and most importantly, the current study employed a pre-post within-subject design without a separate control group (e.g., a no-music condition or an alternative non-musical activity). This limits our ability to definitively attribute the observed neural and behavioral changes solely to the specific effects of music listening, as opposed to non-specific factors such as the passage of time, habituation to the scanner environment, or general relaxation effects. The original dataset on which our analyses are based was designed to explore the neural correlates of a personalized music intervention in FM patients, prioritizing ecological validity through the use of self-selected, highly pleasant music. While this design provides strong preliminary evidence for the association between music listening and altered brain function/pain perception, it cannot establish causality or rule out placebo or expectancy effects.

## Conclusion

The current study provides evidence of music’s role in pain regulation for FM patients, highlighting its positive physical and psychological effects and its impact on brain function. By combining three key RS-fMRI measures – ALFF, ReHo and DC – we examined spatial and temporal connectivity before and after music listening. The findings identified key functional regions in the frontal, temporal, and occipital lobes, as well as the sensorimotor area and cerebellum. These results are of great significance for further explaining the spatial distribution of brain functions and the temporal development characteristics of cognitive processing involved in the neural mechanism of MIA. Future research should incorporate more rigorous experimental controls—such as mock scanner acclimation—and explore dynamic network interactions across multiple brain systems. Additionally, integrating neurochemical assays and longitudinal designs could further clarify the mechanisms of MIA and enhance its translation to personalized, evidence-based clinical practice.

## Data Availability

The datasets presented in this study can be found in online repositories. The names of the repository/repositories and accession number(s) can be found in the article/supplementary material.

## Footnotes

1
https://openneuro.org/datasets/ds001928/versions/1.1.0

2
http://rfmri.org/dpabi

3
https://www.fil.ion.ucl.ac.uk/spm/software/spm12/

4
http://www.restfmri.net
